# Supplementary material for: Counterpropagating topological and quantum Hall edge channels
Source: Nat Commun. 2022 May 13;13:2682. doi: 10.1038/s41467-022-29815-2 (PMC9106760; doi:10.1038/s41467-022-29815-2)
Supplement: Supplementary file 1 — Supplementary Information [file 41467_2022_29815_MOESM1_ESM.pdf]

SUPPLEMENTARY INFORMATION

Counterpropagating topological and quantum Hall edge channels

Saquib Shamim,[1,2,*] Pragya Shekhar,[1,2] Wouter Beugeling,[1,2] Jan Böttcher,[3] Andreas Budewitz,[1,2] Julian-Benedikt Mayer,[3] Lukas Lunczer,[1,2] Ewelina M. Hankiewicz,[3] Hartmut Buhmann,[1,2] and Laurens W. Molenkamp[1,2,†]

[1]*Experimentelle Physik III, Physikalisches Institut, Universität Würzburg, Am Hubland, 97074 Würzburg, Germany*
[2]*Institute for Topological Insulators, Universität Würzburg, Am Hubland, 97074 Würzburg, Germany*
[3]*Institut für Theoretische Physik und Astrophysik, Universität Würzburg, Am Hubland, 97074 Würzburg, Germany*

(Dated: April 19, 2022)

* Saquib.Shamim@physik.uni-wuerzburg.de
† Laurens.Molenkamp@physik.uni-wuerzburg.de

Supplementary Note 1: Magnetotransport in dry-etched devices with SiO_2/Si_3N_4 and HfO_2 gate dielectric

In Supplementary Table 1, we list the devices presented here with their relevant properties, in particular device dimensions and etching method. In Supplementary Figure 1, we provide measurements of the transverse resistance R_{xy} for two devices fabricated from QW4 using the dry-etching process but with different dielectrics: Dev 4.d1 in Supplementary Figure 1a uses SiO_2/Si_3N_4, while Dev 4.d2 in Supplementary Figure 1b uses HfO_2, as the dielectric. For both devices, we see a $\nu = 1$ quantum Hall plateau at high magnetic field (instead of the theoretically expected trivial insulating state), confirming that the observed effect is due to disorder from the etching process and does not depend on the dielectric used.

Supplementary Note 2: The quantum spin Hall effect in (Hg,Mn)Te quantum wells

Supplementary Figure 2a and c show the gate voltage and magnetotransport characteristics of microstructures fabricated from QW5 (9 nm thick with 1.2 % Mn) at 1.4 K. Supplementary Figure 2a shows that we can tune the chemical potential from n- to p-conduction regime by decreasing the gate voltage V_g for Dev 5.w1 and Dev 5.w2. For V_g in the range of -0.1 to -0.25 V, the conductance is quantized close to $2e^2/h$ (dashed line in Supplementary Figure 2a) due to the quantum spin Hall effect. Similar conductance quantization due to the quantum spin Hall effect is also seen in Dev 6.w, fabricated from QW6 (11.5 nm thick with 2.2 % Mn), see Supplementary Figure 2b. The inset of Supplementary Figure 2c shows the conductance quantization for Dev 5.w1 at 20 mK. The long quantized plateau in conductance observed in Supplementary Figure 2b and inset of Supplementary Figure 2c is due to the pinning of the chemical potential to the 'camel back' feature in the valence band (due to band inversion-induced van Hove singularity in the valence band of topological (Hg,Mn)Te quantum wells), as already explained in Ref. 14 of the main text. For Dev 5.w1, at $V_g = -0.2$ V (where the conductance is quantized to $2e^2/h$ at $B = 0$), application of a perpendicular magnetic field (> 160 mT) results in the quantization of the transverse resistance R_{xy} to $-h/e^2$ (solid line in Supplementary Figure 2c) and simultaneously R_{xx} goes to zero. This low field $\nu = -1$ plateau is the emergent quantum Hall plateau, which emerges from the quantum spin Hall edge channels, as discussed previously in Ref. 13 of the main text.

Supplementary Table

Device label	quantum well	thickness d_{QW} (nm)	Mn conc. x	Dimensions $L \times W$ (μm$\times\mu$m)	Fabrication method	V_0 (V)	$R_{xx}^{\max}$ (Ω)	B^* (T)
Dev 4.d1	QW4	7	0	30×10	dry etch	-1.21	84×10^3	2
Dev 4.d2	QW4			30×10	dry etch	-0.37	58×10^3	
Dev 5.w1	QW5	9	1.2%	1.9×1.7	wet etch	-0.1	13×10^3	4.9
Dev 5.w2	QW5			3×4	wet etch	-0.15	16×10^3	
Dev 6.w	QW6	11.5	2.2%	1×5.5	wet etch	-1	13×10^3	4.3

Supplementary Table 1. Devices presented in the supplementary information with device labels, quantum well number, thickness and Mn concentration, Hall bar dimensions, fabrication method (dry or wet etching), V_0 (voltage for maximal longitudinal resistance), $R_{xx}^{\max}$ (maximal longitudinal resistance at $B = 0$) and B^* (magnetic field above which the band inversion is lifted). The values are given at temperature $T = 1.4\,\mathrm{K}$ for devices Dev 4.d1, Dev 4.d2, Dev 5.w2 and at $T = 20\,\mathrm{mK}$ for Dev 5.w1 and Dev 6.w.

Supplementary Figures

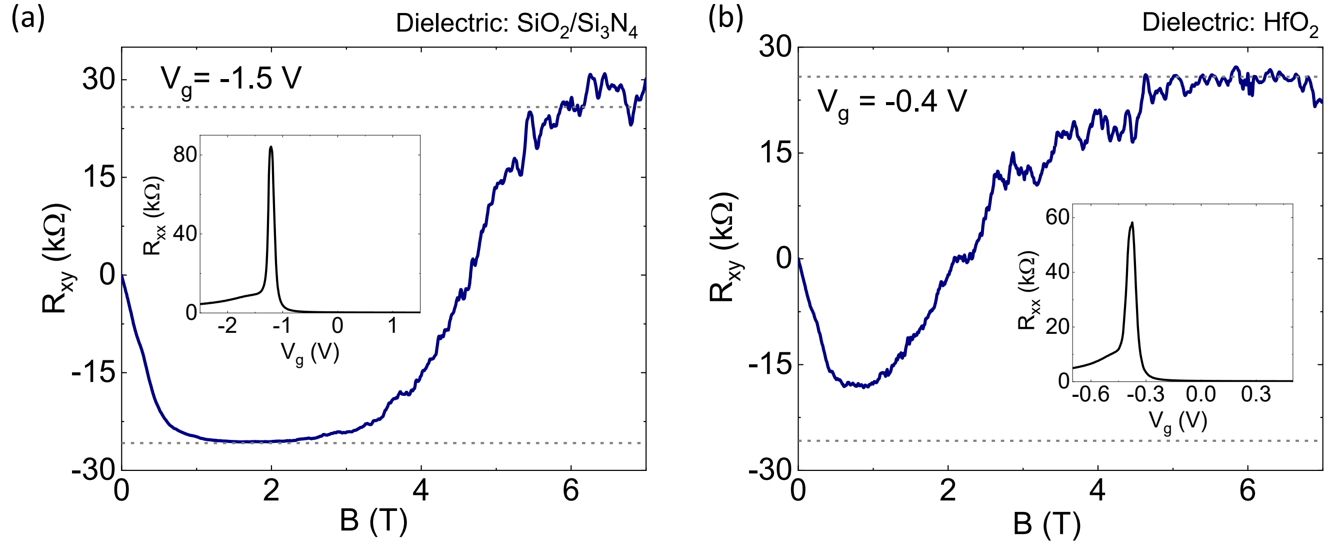

Supplementary Figure 1: Comparison of devices fabricated from QW4 (7 nm HgTe quantum well) with SiO_2/Si_3N_4 **and** HfO_2 **dielectric.** The transverse resistance R_{xy} as a function of magnetic field at 1.4 K for **a,** Dev 4.d1, which uses SiO_2/Si_3N_4 as dielectric, at gate voltage $V_g = -1.5\,V$. **b,** Dev 4.d2, which uses HfO_2 as dielectric at $V_g = -0.4V$. The insets show the longitudinal resistance R_{xx} as a function of V_g.

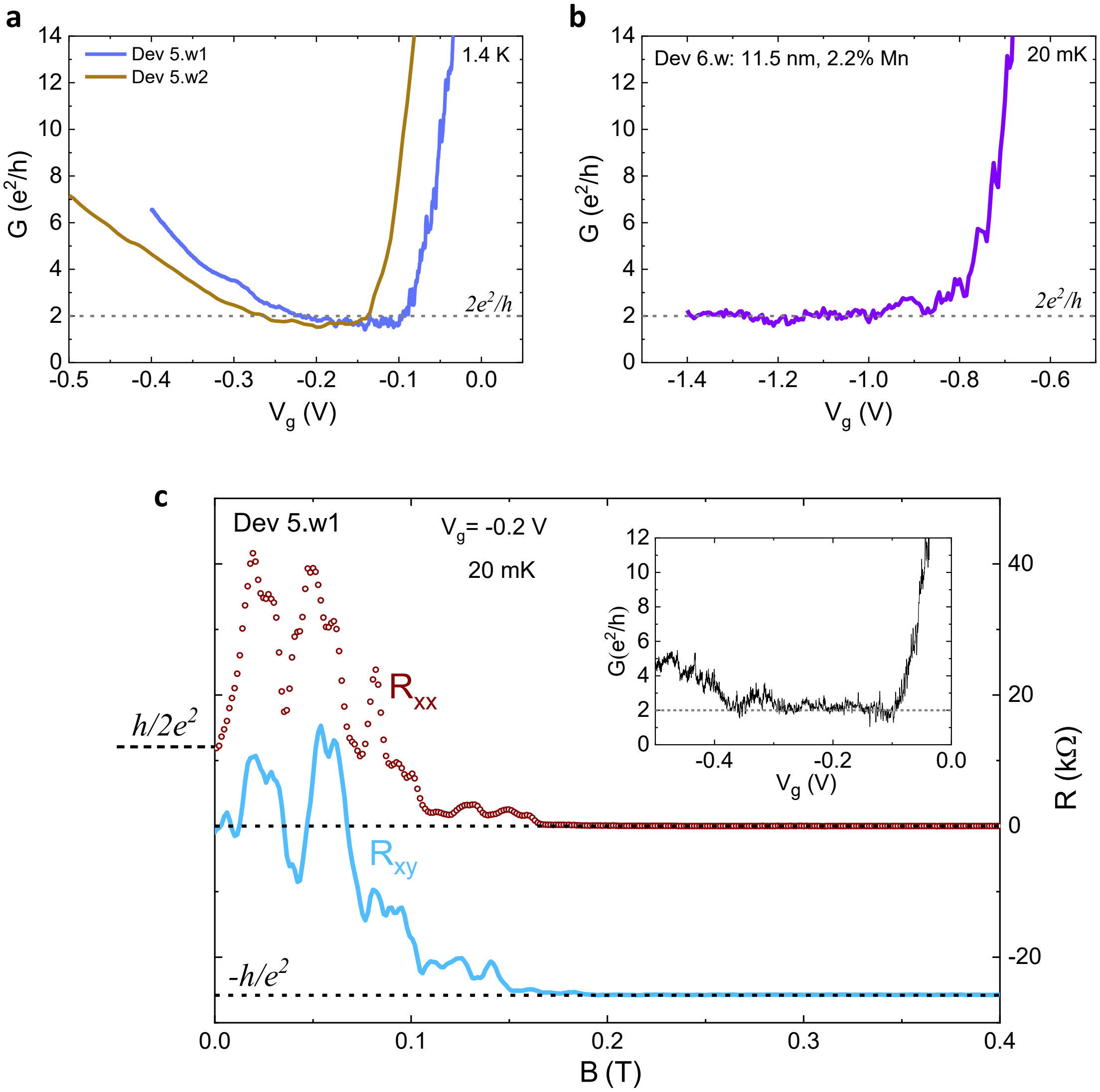

Supplementary Figure 2: Quantized conductance in (Hg,Mn)Te quantum wells and transition to the emergent quantum Hall effect. The conductance G, as a function of gate voltage V_g for **a,** Dev 5.w1 and Dev 5.w2 at 1.4 K. **b,** Dev 6.w at 20 mK. **c,** Longitudinal (open circles) and transverse resistance (solid line), R_{xx} and R_{xy} respectively of Dev 5.w1, as a function of magnetic field B at 20 mK for $V_g = -0.2$ V. The inset shows G as a function of V_g.

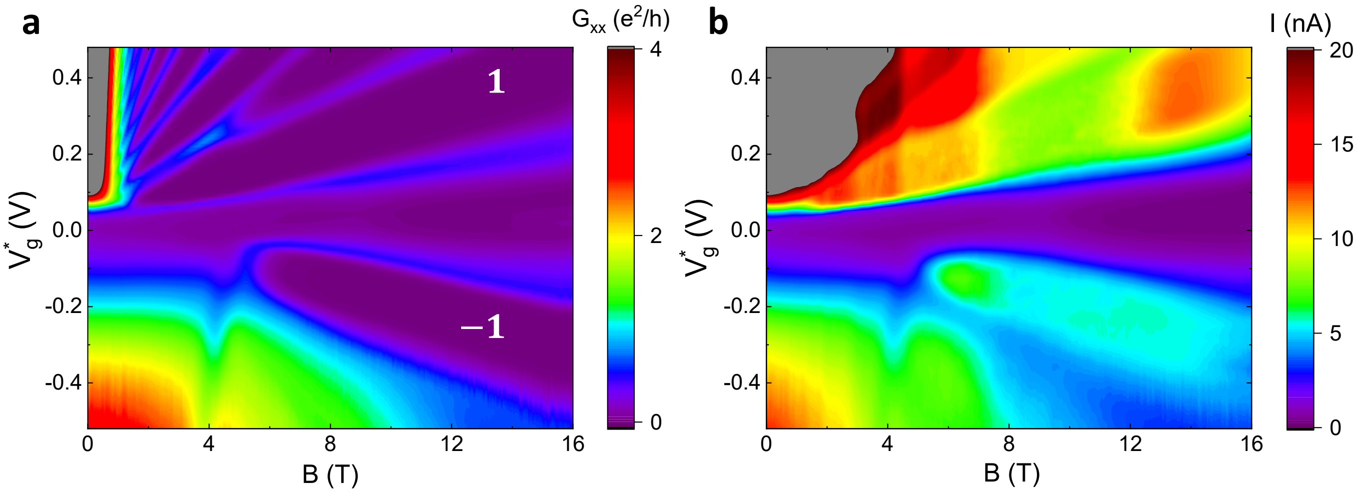

Supplementary Figure 3: Magnetotransport for wet-etched device Dev 2.w. a, Color plot of the longitudinal conductance G_{xx} as a function of B and V_g^*. **b,** Color plot of the current I flowing through the device as a function of B and V_g^*.
